# Comparative Analysis between Wild and Cultivated Cucumbers Reveals Transcriptional Changes during Domestication Process

**DOI:** 10.3390/plants9010063

**Published:** 2020-01-03

**Authors:** Eslam M. Abdel-Salam, Mohammad Faisal, Abdulrahman A. Alatar, Quaiser Saquib, Hend A. Alwathnani

**Affiliations:** 1 Department of Botany & Microbiology, College of Science, King Saud University, P.O Box 2455, Riyadh 11451, Saudi Arabia; eabdelsalam@ksu.edu.sa (E.M.A.-S.); aalatar@ksu.edu.sa (A.A.A.); wathnani@ksu.edu.sa (H.A.A.); 2 Zoology Department, College of Science, King Saud University, P.O Box 2455, Riyadh 11451, Saudi Arabia; quaisersaquib0@gmail.com

**Keywords:** transcriptomics, *Cucumis sativus*, transcription factors, gene ontology, evolutionary changes

## Abstract

The cultivated cucumber (*Cucumis sativus* L.) was reported to have been developed from a wild cucumber (*Cucumis hystrix* Chakrav.), nevertheless, these two organisms exhibit noteworthy differences. For example, the wild cucumber is known for its high resistance to different biotic and abiotic stresses. Moreover, the leaves and fruits of the wild cucumber have a bitter taste compared to the cultivated cucumber. These differences could be attributed mainly to the differences in gene expression levels. In the present investigation, we analyzed the RNA-sequencing data to show the differentially expressed genes (DEGs) between the wild and cultivated cucumbers. The identified DEGs were further utilized for Gene Ontology (GO) and pathway enrichment analysis and for identification of transcription factors and regulators. In the results, several enriched GO terms in the biological process, cellular component, and molecular functions categories were identified and various enriched pathways, especially the biosynthesis pathways of secondary products were recognized. Plant-specific transcription factor families were differentially expressed between the wild and cultivated cucumbers. The results obtained provide preliminary evidence for the transcriptional differences between the wild and cultivated cucumbers which developed during the domestication process as a result of natural and/or artificial selection, and they formulate the basis for future genetic research and improvement of the cultivated cucumber.

## 1. Introduction

Cultivated cucumber (*Cucumis sativus* L.) is one of the most important cultivated crops around the world. Its production in 2016 exceeded 80.6 million tons, the average yield was 375,893 kg/ha, and the total harvested area reached 2,144,672 ha, ranking its worldwide production at the 18th position among all edible crops [1]. For the last 5 years, China has been considered as the largest producing country of cucumbers. Cucumber is one of the most widely cultivated vegetable crops, after tomato and onion [2]. Furthermore, cucumber is one of the notable sources of vitamin K (100 g of cucumber could serve around 16% of the adult’s daily needs). Therefore, morphological, physiological and genetic characteristics of the cultivated cucumber have been studied extensively. The complete genome of the cultivated cucumber (‘Chinese long’ inbred line 9930, which is commonly used in modern cucumber breeding) was published in 2009 [3]. With a length of 243.56 Mb, around 26,682 genes were predicted using three different gene-prediction methods (i.e., cDNA-expressed sequence tag (EST), homology based and ab initio), with an average of 4.39 exons and a mean coding sequence size of 1046 bp per gene. Moreover, besides coding genes, 699 tRNA, 292 rRNA fragments, 192 small nuclear RNA, 238 small nucleolar RNA and 171 miRNA genes were identified in the cucumber genome [3]. The predicted genes belong to 15,669 gene families (including 4362 that are cucumber unique families of which 3784 are single-gene families) and are located on seven different chromosomes. 

Phenotypic differences between wild and cultivated plants are generally apparent and have been studied extensively in many plant species. Genetic mechanisms underlying those phenotypic differences and the domestication processes were studied in several plants including rice [4], tomato and pepper [5], and wheat, maize, and sunflower [6]. The studies revealed that the changes in transcription levels are continuous during the domestication process of these plants, and they provided sufficient evidence about the relationship between phenotypic differences and genetic alterations. Nevertheless, in several cases, the differences between wild and cultivated plants are attributed to a small number of genetic loci. However, no studies examined the transcriptional changes during the domestication process of cucumber. Application of comparative transcriptome analysis to reveal changes during the domestication process and to capture transcriptional variations that could be utilized in breeding programs is well-established and applied in several plant species including chia [7] and eggplant [8]. Comparative transcriptome analysis of wild and cultivated watermelon led to the identification of several differentially expressed genes (DEGs) in the flesh and mesocarp of cultivated and wild watermelon [9]. Enriched pathways in the tissues of cultivated watermelon were mainly related to fruit quality such as sweetness and flavor; on the other hand, the pathways enriched in the tissues of wild watermelon were related to abiotic stress. In tomato, 126 up-regulated and 87 down-regulated DEGs were identified via analyzing the transcriptome of wild and cultivated plants. These genes were related to abiotic resistance especially salinity and drought [10]. 

Changes happening during domestication may include alteration in stress tolerance/resistance, phenotypic differences and/or genetic variations. Wild cucumber (*C. hystrix*) plants are known to have increased tolerance to different abiotic stresses including salinity, heat, cold, and shading as compared to cultivated plants [11]. Leaves of wild cucumber are characterized by bitter taste and smaller size as compared to cultivated cucumber plants, and fruits of the cultivated cucumber and its wild relative *Cucumis hystrix* Chakrav. differs in shape and taste (bitterness) [12]. The main contributor to the bitter taste in both leaves and fruits is cucurbitacin (CuC), a secondary product used for plants’ defense against herbivores and has useful medicinal effects including hepatoprotective and anti-tumor properties [13,14]. Studies on the biosynthesis pathway of CuC revealed the genetic basis of cucumber domestication. Bitterness (*Bi*) was identified as one of the essential genes in the biosynthesis of CuC [12]. Two different genes are responsible for regulation of *Bi* gene expression, namely bitter leaf (*Bl*) and bitter fruit (*Bt*). These genes have the ability to directly bind with E-box elements in the *Bi* promoter leading to the regulation of *Bi* gene expression and, thus regulation of CuC biosynthesis [12]. However, information about the genetic differences between wild and cultivated cucumbers is lacking. The current study is considered the first to examine the differences in transcriptome of wild and cultivated cucumbers. In the current study, we used RNA sequencing (RNA-Seq) data obtained from online repositories to examine the transcriptional differences and the evolution of tissue-specific transcriptomes between *C. hystrix* and *C. sativus* plants. We selected *C. hystrix* for this analysis because of its potential as a genetic donor in breeding and genetic improvement programs of cultivated cucumbers. 

## 2. Results

### 2.1. Data Alignment

Sixteen different transcriptome reads, representing three replicates for leaves and roots of *C. hystrix* (wild cucumber) and five replicates for the leaves and roots of *C. sativus* (cultivated cucumber), were mapped to the cucumber reference genome using the HISAT2 alignment software (Table 1). The alignment rates of clean reads ranged from 70.41% to 76.28% in *C. hystrix*, with multiple alignments rate ranging from 1.35% to 3.36%. In *C. sativus*, alignment rates ranged from 90.36% to 97.49% with multiple alignment rates ranging from 1.33% to 2.47%. The average alignment rate for all *C. hystrix* samples was 73.52% as compared to the average alignment rate for all *C. sativus* samples of 94.61%. This variation in alignment rates could be attributed to using the cultivated cucumber genome as reference genome for alignment and further comparisons. Therefore, more rigid rules were followed to identify differentially expressed genes as elaborated in the methods section.

**Figure 1:**
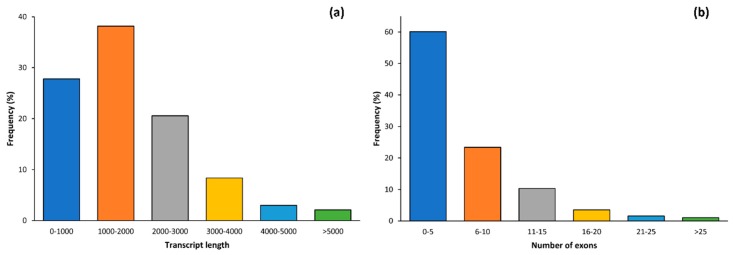
Distribution of length (**a**) and number of exons (**b**) of assembled transcripts in *C. hystrix* and *C. sativus*.

A total of 104,921 transcripts were obtained after mapping the cleaned reads to the cultivated cucumber reference genome. Of these, 29,172 transcripts (27.80%) were shorter than 1000 bp, and only 2197 transcripts (2.09%) were longer than 5000 bp. In general, the majority (65.96%) of transcript lengths were below than 2000 bp. Furthermore, 90,804 transcripts which accounts for (86.54%) of all assembled transcripts had lengths less than 3000 bp (Figure 1a). Number of exons in all the assembled transcripts were examined and the obtained results showed that 60.11% (63,067 transcripts) of the assembled transcripts had five exons or less. Only 6.17% of the assembled transcripts had more than 15 exons. (Figure 1b).

### 2.2. Identification of Differentially Expressed Genes (DEGs)

**Figure 2:**
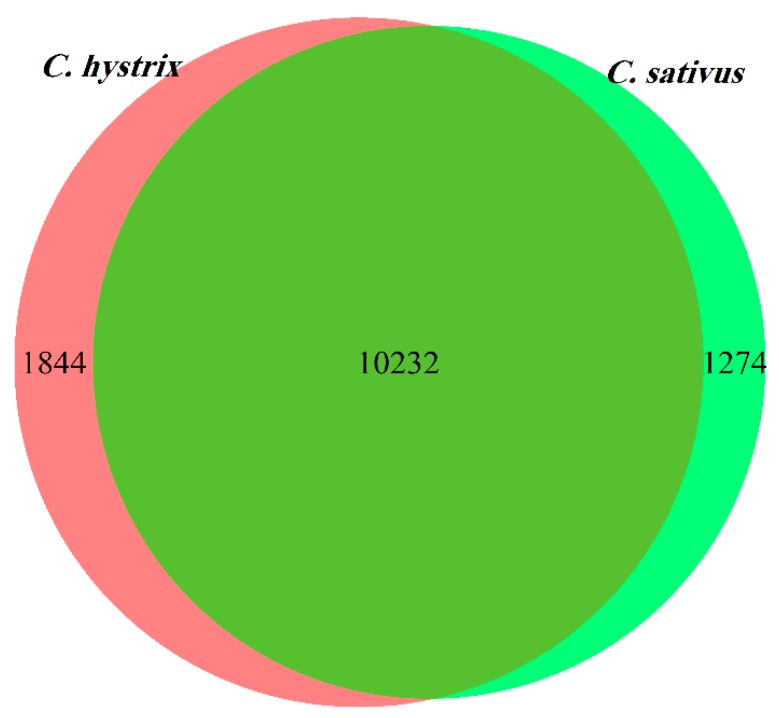
Gene expression map of cultivated cucumber (*C. sativus*) and its wild relative *C. hystrix*.

The classification and quantification of expressed transcripts and genes in the two cucumber species identified 11,506 genes expressed in *C. sativus*, 12,076 genes expressed in *C. hystrix*, and 10,232 genes expressed in both plant species (Figure 2). The number of genes expressed in both species accounted for 88.93% and 84.73% of the expressed genes in *C. sativus* and *C. hystrix*, respectively, indicating the high genetic similarity between the two species.

**Figure 3:**
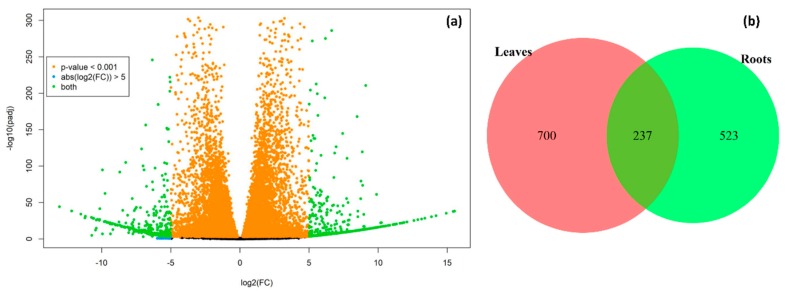
(**a**) Volcano plot showing the map of differential gene expression analysis and (**b**) Venn diagram illustrating number of differentially expressed genes (DEGs) identified in leaves and roots of *C. sativus* and *C. hystrix* cucumber.

**Figure 4:**
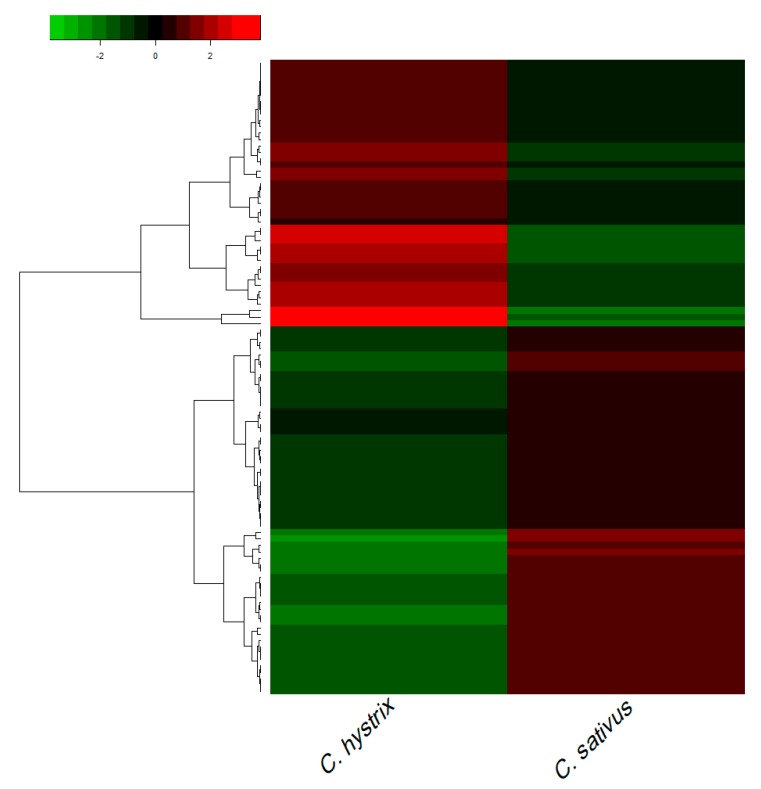
Heatmap showing the average expression levels of selected genes in leaves and roots of the *C. sativus* and *C. hystrix*.

DEGs between the two studied plants were identified and selected for further analysis using featureCounts and DESeq2 packages in R based on the filtering rule (genes with *p* < 0.001 and abs (log2 fold change) > 5.0) (Figure 3a). The expression of several genes was down-regulated in *C. sativus* and up-regulated in *C. hystrix* and vice versa. Differential expression analysis of leaves and roots sequences from *C. hystrix* and *C. sativus* identified 937 and 760 DEGs in leaves and roots, respectively, with 237 genes identified as DEGs in both tissues (Figure 3b). The heatmap illustrated in Figure 4 shows the changes in expression pattern of the top 100 DEGs (based on adjusted p-values) in *C. sativus* and *C. hystrix* studied tissues. A full list of identified DEGs in leaves and roots of *C. sativus* and *C. hystrix* is provided as Supplementary Tables (S1 and S2). We hypothesized that these DEGs might be related to changes happening during domestication and/or stress tolerance/resistance. Further analysis helped to reveal their potential roles.

### 2.3. Gene Ontology (GO) Analysis of DEGs

**Figure 5:**
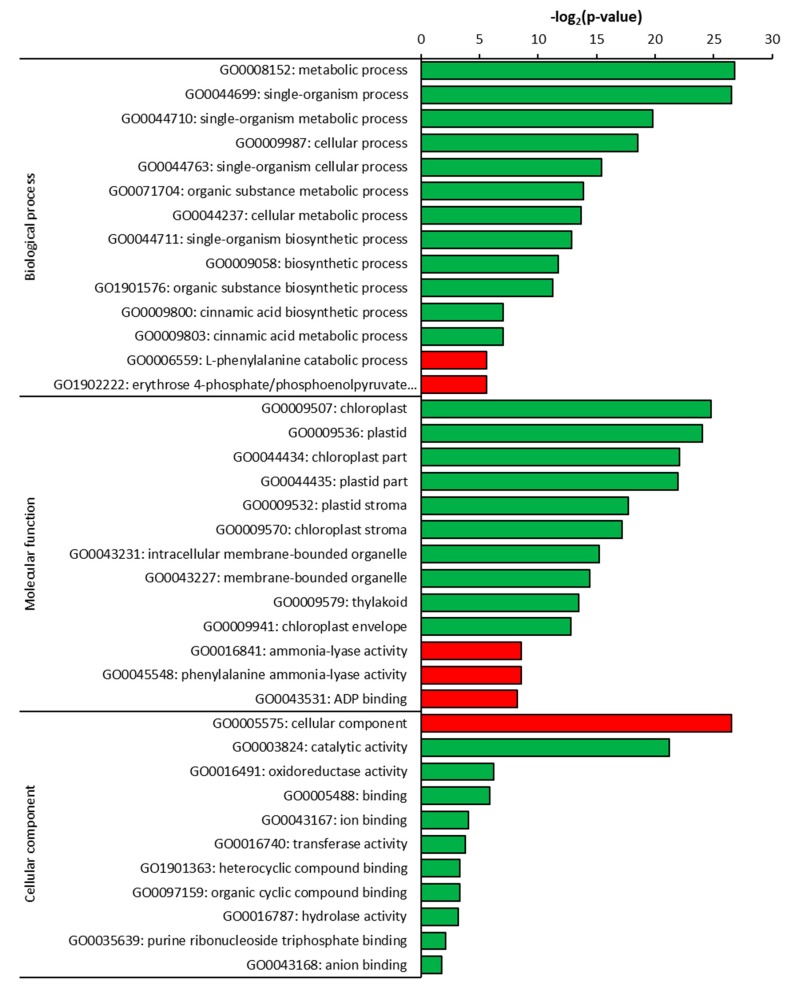
Gene Ontology (GO) enrichment analysis of differentially expressed genes in leaves of *C. sativus* as compared to *C. hystrix*. Green bars show down-regulated and red bars show up-regulated GO terms.

The classification of up- and down-regulated DEGs in *C. sativus* as compared to its wild relative *C. hystrix* was conducted by searching the identified genes against the Gene Ontology (GO) database available on the Cucurbit Genomics Database (CuGenDB) website with three different categories, namely biological process, cellular component, and molecular function. In leaves, the most down-regulated biological processes enriched in *C. sativus* were metabolic process (GO0008152) and single-organism process (GO0044699) (Figure 5). Cellular component GO terms of cellular component category was the only term enriched in the up-regulated genes in *C. sativus*; however, several molecular function terms were enriched by down-regulated genes in *C. sativus* including catalytic activity and oxidoreductase activity. Similarly, ammonia lyase activity (GO0016841), phenylalanine ammonia-lyase activity (GO0045548) and ADP binding (GO0043531) were the most enriched pathway in the category of molecular function by up-regulated DEGs identified in leaves of *C. sativus* as compared to its wild relative *C. hystrix*. Several GO terms in the same category were enriched by down-regulated DEGs identified in *C. sativus* as compared to *C. hystrix*, most of them related to the chloroplast metabolic processes.

**Figure 6:**
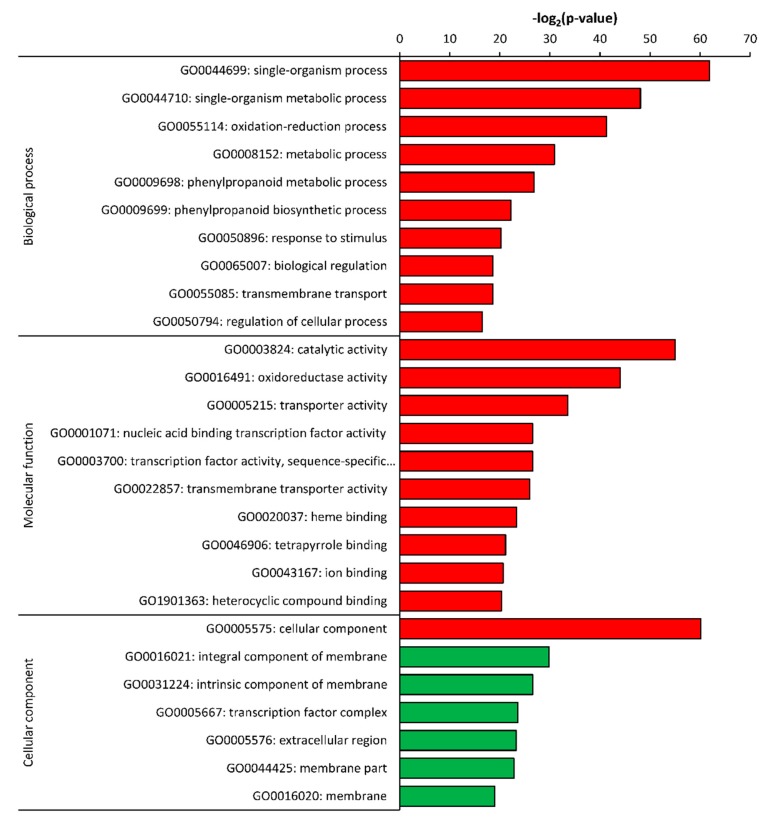
Gene Ontology enrichment analysis of differentially expressed genes in roots of *C. sativus* as compared to *C. hystrix*. Green bars show down-regulated and red bars show up-regulated GO terms.

Up- and down-regulated DEGs identified in roots of *C. sativus* as compared to its wild relative *C. hystrix* were also searched against the GO terms database to identify the terms enriched by both group of genes (Figure 6). The analysis showed that no GO terms enriched by down-regulated genes in biological process and molecular function categories. However, only the cellular component term in cellular component category was enriched by down-regulated genes. The most enriched GO terms by up-regulated genes included single-organism process and oxidation-reduction process (GO005114) in the biological process category in addition to catalytic activity and oxidoreductase activity in the molecular function category. Integral (GO0016021) and intrinsic (GO0031224) components of membrane were the highest enriched terms in the cellular component category by up-regulated genes in *C. sativus*. 

### 2.4. Pathway Enrichment Analysis of DEGs

**Figure 7:**
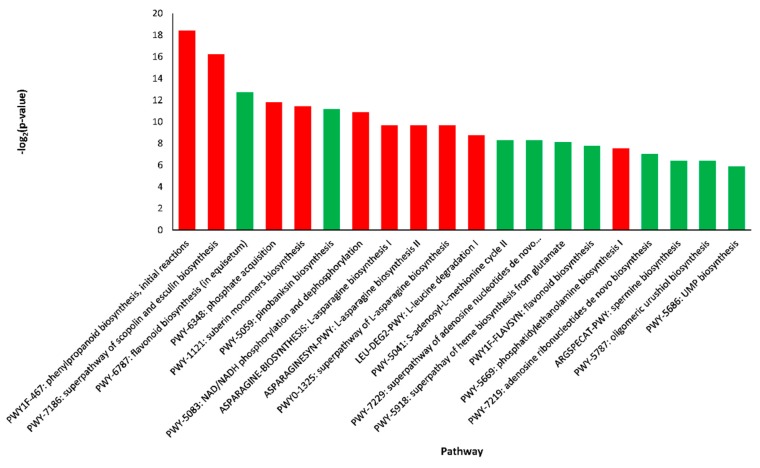
Pathway enrichment analysis of differentially expressed genes in leaves of *C. sativus* as compared to *C. hystrix*. Green columns show down-regulated and red columns show up-regulated pathways.

The DEGs identified in leaves and roots of the *C. sativus* as compared to *C. hystrix* plants were searched against the cucumber Chinese Long v 2.0 pathway database on CuGenDB website to identify the enriched pathways. Figure 7 shows the top 10 pathways enriched by up-regulated (red columns) and down-regulated (green columns) DEGs. Interestingly, the most enriched pathways by up-regulated genes in *C. sativus* included the superpathway of scopolin and esculin biosynthesis (PWY-7186). On the other hand, the pathways enriched by down-regulated genes in *C. sativus*, surprisingly, included flavonoid biosynthesis (PWY-6787) and pinobanksin biosynthesis (PWY-5059). 

**Figure 8:**
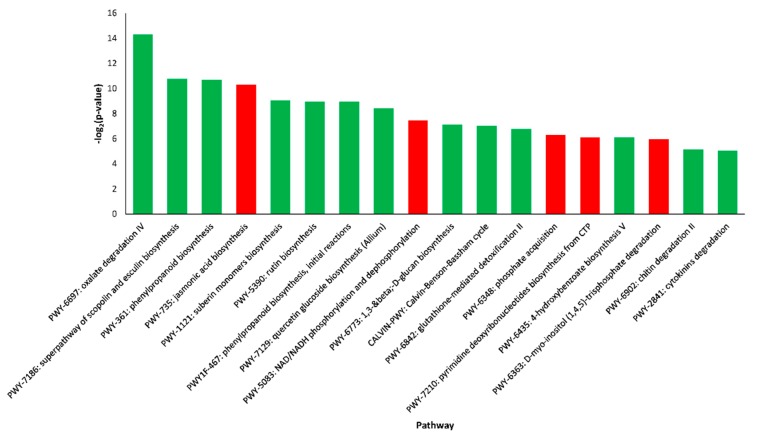
Pathway enrichment analysis of differentially expressed genes in roots of *C. sativus* as compared to *C. hystrix*. Green columns show down-regulated and red columns show up-regulated.

Searching the DEGs identified in the roots of *C. sativus* as compared to *C. hystrix* against pathway database revealed 13 pathways enriched by down-regulated genes and 5 enriched by up-regulated genes (Figure 8). In contrast to leaves, the superpathway of scopolin and esculin biosynthesis were enriched by down-regulated genes. The pathways enriched by down-regulated genes in roots of *C. sativus* included, interestingly, the jasmonic acid biosynthesis (PWY-735) pathway.

### 2.5. Identification of Differentially Expressed Transcription Factors (TFs) and Transcriptional Regulators (TRs)

**Figure 9:**
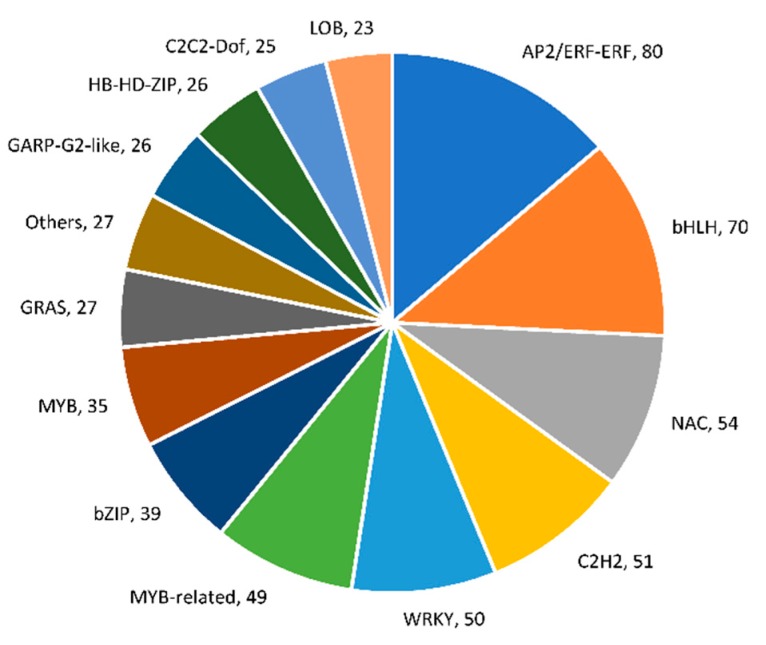
The most represented families of transcription factors and transcription regulators (family name, number of represented genes) of differentially expressed genes in *C. sativus* as compared to *C. hystrix*.

The identified DEGs in the tissues of *C. sativus* plants as compared to *C. hystrix* were searched against transcription factor (TF) and transcriptional regulator (TR) families in Cucurbitaceae plants. The most represented TF families were apetala2/ethylene responsive factor (AP2/ERF) family with 80 DEGs, followed by the basic helix-loop-helix (bHLH) family with 70 DEGs, the NAC family with 54 DEGs, the C2H2 Zinc Finger family with 51 DEGs, and the WRKY family with 50 DEGs (Figure 9). 

## 3. Discussion

Examining the transcriptome variations between cultivated crops and their wild relatives is one of the most promising areas of research as it reveals the genetic mechanisms of trait variations related to changes happening during domestication and stress tolerance/resistance of those plants. The results of the current study showed that the length of the majority of the assembled transcripts (86.54%) was less than 3000 bp and more than 83.49% of the assembled transcripts had less than 10 exons (60.11% of the transcripts had less than 5 exons). The average length of the transcribed (N50) region of the genes in *C. sativus* is 3058 bp [15] and the average number of exons per gene is 5.49 [15].

*C. hystrix* was confirmed as the closest wild relative of *C. sativus* using chloroplast and nuclear DNA sequences from 100 different *Cucumis* accessions [16]. Similarly, in the current study, the analysis of gene expression showed that 88.93% and 84.73% of the expressed genes in cultivated and wild cucumber, respectively, were expressed in both plants, thus supporting the close genetic similarity between these two species. *C. hystrix* and *C. sativus* are thought to be the only *Cucumis* species native to Asia [2]. Another phylogenetic study based on 79 chloroplast and 20 internal transcribed spacer (ITS) sequences corroborated the cultivated cucumber’s sister relationship with *C. sativus* [2]. Analysis of genetic expression performed in the current study supported the findings obtained by several phylogenetic studies showing the close relationship between *C. sativus* and its wild relative (*C. hystrix*) [16,17,18,19,20,21].

The results obtained in this study identified 937 and 760 DEGs in leaves and roots, respectively, with 237 genes identified as DEGs in both tissues which indicated several differences in genetic expression between *C. sativus* and *C. hystrix* plants. Our further analysis was based on the hypothesis that those genes might relate to processes that happened during domestication including loss of bitterness, changes in nutritional value and changes in response to different abiotic stresses. In tomato, 126 and 87 up- and down-regulated DEGs were identified and most of them were related to drought, salt resistance and nutrition [10]. Similarly, DEGs identified in the roots of wild and cultivated carrot plants were mainly related to the development of the storage root [22]. Enrichment analysis of DEGs identified in wild and cultivated watermelon revealed that most of these genes were related to metabolic pathways and biological processes [9]. In rice, roots of wild plants were more resistant to *Magnaporthe oryzae* fungal infection and the enrichment analysis showed that genes related to the phenolic and terpenoid pathways and the phenolic and terpenoid syntheses-related mevalonate pathway (the pathways which adversely affected by the fungal infection) were more enriched in wild rice plants as compared to cultivated ones [23].

The identified DEGs were subjected to functional analysis using GO and pathway enrichment analyses for identification of their potential roles in changes happening during domestication and/or stress resistance. The results obtained showed a significant enrichment of the biological process, cellular component, and molecular function categories in leaves and roots of *C. sativus* as compared to its wild relative *C. hystrix*. The distribution of DEGs and the distribution of GO and pathway enrichment in the different tissues of the two cucumber species might provide evidence for the phenotypic differences between these studied plants. In leaves of *C. sativus* plants, the biosynthesis of cinnamic acid were significantly down-regulated as compared to *C. hystrix*. Cinnamic acid is one of the most famous autotoxin found in cucumber and it has an adverse effect on plant morphogenesis and development via inhibitory regulation [24]. Therefore, changes in regulation of biosynthesis of cinnamic acid could be considered as an indicator for the domestication process and selection for the loss of bitterness, a property related to the biosynthesis of cucurbitacin, in the cultivated cucumber [12]. Furthermore, our results showed that the *Csa5G156220* gene was up-regulated 7-fold in *C. sativus* as compared to *C. hystrix* (S1). It was proved that *Csa5G156220* gene regulates the expression of the *Bi* gene which in turn controls the bitter phenotype in the whole plant. Increasing *Csa5G156220* expression increased the expression of the *Bi* gene and stimulated the non-bitter phenotype in cucumber plants [12]. *Bi* gene is co-expressed with three more genes (*Csa6G088160*, *Csa6G088170* and *Csa6G088700*) in controlling the non-bitter phenotype. Our results showed that expression of these genes was up-regulated in *C. sativus* leaves by more than 5-fold each as compared to their expression in *C. hystrix* leaves (S1). In contrast, the identified up-regulated DEGs in roots of *C. sativus* plants as compared to *C. hystrix* enriched mainly GO terms and metabolic pathways related to redox activity including oxidoreductase enzymes activity, which reflects the increased tolerance of *C. hystrix* to different abiotic stresses including salinity, heat, cold, and shading [11]. Moreover, changes in the biosynthesis of jasmonic acid were observed via pathway analysis. Jasmonic acid is one of the major phytohormones that stimulates plant response to abiotic stress [23]. Our results showed that biosynthesis of jasmonic acid were down-regulated in *C. sativus* plants as compared to its wild relative *C. hystrix*, which might interpret the known differences in stress tolerance/resistance between cultivated cucumber and its wild relative [11].

The query of DEGs against different families of transcription factors and regulators identified the most represented TF families, which included AP2/ERF (80 genes), bHLH (70 genes), NAC (54 genes), C2H2 (51 genes), and WRKY (50 genes). These TF families are highly expressed in cultivated cucumber cultivars with pivot roles in traits with relation to domestication including loss of bitter taste [25]. For example, the *CsERF* (*Csa7G448110*) gene, a member of AP2/ERF, has been reported to regulate the expression of the *Bi* gene responsible for bitter taste in both leaves and fruits [26]. In the current study, expression of this gene was up-regulated in *C. sativus* as compared to *C. hystrix* which may partially explain the phenotypic differences between *C. sativus* and *C. hystrix* plants. The NAC TF family is one of the largest plant-specific families, with around 167 members in the banana, 149 members in rice, 106 members in *Arabidopsis*, and 91 members in the cucumber [27]. Several NAC members were identified to be involved in drought tolerance and adaptation in maize using expression-profiling analysis and genome-wide survey [28]. In addition to their roles in the plant responses to different abiotic stresses, NAC members play significant roles in several biological processes [29]. It was noted that NAC members have different roles in fundamental developmental and biological processes including embryo and shoot apical meristem development, cell wall biosynthesis, floral morphogenesis, and formation of lateral roots [30,31,32,33]. In cucumber, several NAC members have been reported to have fundamental roles response to abiotic stresses [27]. The current study showed that 54 members of NAC family were differentially expressed in the tissues of *C. sativus* and *C. hystrix* which partially explain the differences in these plant responses to abiotic stresses. Similarly, the WRKY TF family is one of the largest TF families in higher plants. Its members play significant roles in plant responses to biotic and abiotic stresses via regulation of hormone signal transduction pathways [34]. In cucumber, about 55 WRKY TFs were identified [35]. We showed that 50 WRKY genes were differentially expressed between *C. sativus* and *C. hystrix* plants. These results might explain the increased resistance of *C. hystrix* plants to different biotic and abiotic stresses as compared to *C. sativus*. The results obtained in the current study provide a clear base for the cultivation and genetic manipulation of cucumber.

## 4. Materials and Methods 

### 4.1. Data Resources

The data used in this study were downloaded from the National Center for Biotechnology Information (NCBI) Sequence Read Archive (SRA) database [36]. All the data available for cultivated cucumber (*C. sativus*) and its wild relative (*C. hystrix*) on the NCBI SRA database were downloaded and quality checked. All the data with good quality were used for further analysis. Collected data were obtained at the trifoliate stage from plants grown in greenhouse (25 ± 2 °C, 16 h photoperiod) without any treatment. Leaf and root transcriptome data of the wild and cultivated cucumbers were obtained from 16 samples and they comprised a total of 308,726,882 reads.

### 4.2. Data Analysis

#### 4.2.1. Quality Check

Quality and adapter trimming of RNA-Seq raw reads was conducted using the Sickle and Trimmomatic software [37,38]. Most of the next-generation sequencing technologies tend to produce reads with lower quality towards the 3` ends. Therefore, sickle software was used to trim the low-quality 3`-end calls which defined based on an average quality of sliding window along the read. The average quality was set to 12 as recommended by the previous studies. For further trimming of adapters and low-quality reads, Trimmomatic software v0.39 were used with default values. After trimming and cleaning, further quality check of the processed transcriptome data was performed using the FastQC program [39]. FastQC software v 0.11.8 provides a set of model analyses which gives a general impression about the RNA-Seq data quality to discover whether data have any problems that need to be handled before further analysis. Reads with a quality score of at least 20 that contained more than 95% of bases remained were retained.

#### 4.2.2. Differential Expression Analysis

The clean raw RNA-Seq reads were aligned to the cucumber reference genome using Hisat2 aligner v2.1.0 [40]. The sequence (fasta files) of cucumber (Chinese Long) cv. 9930 v2 genome was downloaded from the CuGenDB [41] and used as a reference genome for read mapping. Hisat2 is one of the most famous aligners as it is fast and sensitive aligner using a large set of graph FM indices in a new indexing scheme called the “Hierarchical Graph FM index” which allows rapid and accurate alignment of sequencing reads to the reference genome. SHRiMP [42] and GSNAP [43] aligners were, also, utilized to align reads to reference genome for verification of alignment quality. No significant differences were observed between different alignment. Differentially expressed genes were determined using the counting method. In this regard, featureCounts tool v1.6.5 [44] from the Rsubread package [45] was used to count read counts per gene and calculate the gene abundance of the cleaned reads. This tool is ultra-fast and highly efficient and works with both single- and paired-end reads. Afterwards, the count matrix produced by featureCounts tool were normalized and tested for differential expression analysis using DESEq2 package v1.24.0 [46] on R software v3.6.1. All the analysis steps were undertaken using R Studio software v1.2.1335. DESeq2 package depends upon shrinkage estimation for dispersions and fold changes to analyze differences in gene expression between compared groups. For the purpose of differential expression analysis between very closely related species (e.g., *C. hystrix* and *C. sativus*), cross-mapping of reads to a single genome produces fewer false positives as compared to self-mapping [47]. However, specificity of the analysis might be reduced with increased divergence between studied species. To avoid such caveats, we applied controls on false discovery rate using the method described by Benjamini and Yekutieli [48] with a nominal α of 0.05. Furthermore, rigid roles in identifying DEGs were followed via adopting adjusted p-value (*p*) and higher fold change in gene expression. Genes with *p* ≤ 0.001 and log2 fold change > 5.0 were considered DEGs and used for further analysis.

#### 4.2.3. Gene Ontology and Pathway Enrichment Analysis

Gene ontology (GO) terms are a way to unify the representation of the attributes of genes and their products across all living species [49]. The Cucurbit Genomics Database [41,50] is considered a central port for all species from the Cucurbitaceae family. Therefore, we used the tools available on CuGenDB for the identification of enriched GO terms in the two studied plant species. Up-regulated and down-regulated DEGs were used separately for identification of GO term and pathway enrichment analysis. In this regard, the GO term enrichment analysis tool available on the website was implemented using the Cucumber (Chinese Long) v2 database [51] with false discovery rate as the *p*-value correction method and 0.05 as a cutoff *p*-value for significantly enriched GO terms. The significantly enriched pathways were examined using the pathway enrichment analysis tool available on CuGenDB website based on the Cucumber (Chinese Long) v2 database with a cutoff *p*-value of 0.05. 

#### 4.2.4. Gene Ontology and Pathway Enrichment Analysis

All DEGs were queried against TF and TR families using the batch query tool available on the CuGenDB website. Thereafter, an in-house python script (available upon request) was used to group the genes from the same family and count each group. The script reported the number of unique families, their names, and the percentage of genes in each family.

## 5. Conclusions

In the current study, the transcription patterns of the cultivated cucumber and its wild relative were compared using RNA-Seq data. The results obtained confirm the genetic similarity between the cultivated cucumber (*C. sativus*) and its wild relative (*C. hystrix*). Moreover, the results obtained using differential expression and GO and pathway enrichment analyses revealed various significant features of the wild and cultivated cucumbers. It was observed that the majority of up-regulated genes in cultivated cucumber have relations to processes happening during domestication including loss of bitterness in leaves; however, down-regulated genes in general were classified in response to stress categories. These results lay the foundation stone for further research and annotation to reveal changes in the transcriptome of wild and cultivated cucumbers. Such results could be utilized and would greatly assist in breeding and genetic enhancement programs of the cultivated cucumber. However, further research is needed to improve knowledge about the domestication process of the cucumber and genetic differences between the wild and cultivated cucumber plants.

## Figures and Tables

**Table 1:** Alignment summary of leaf and root transcriptome datasets for *C. hystrix* and *C. sativus*.

Accession No.	Organism	Tissue	Total Reads	Mapped Reads		Multiple Alignments	
				No.	%	No.	%
SRR6375807	*C. hystrix*	Leaves	11,450,746	8,062,837	70.41	385,210	3.36
SRR6375808			11,604,057	8,322,444	71.72	239,315	2.06
SRR6375809			16,732,213	12,047,006	72.00	225,548	1.35
SRR6375813		Roots	13,364,553	10,194,458	76.28	270,044	2.02
SRR6375814			12,118,277	9,042,523	74.62	263,423	2.17
SRR6375815			12,513,487	9,523,603	76.11	249,899	2.00
SRR6854681	*C. sativus*	Leaves	22,850,332	22,251,604	97.38	344,846	1.36
SRR6854682			22,850,332	22,240,800	97.29	308,372	1.35
SRR6854683			22,546,871	21,980,994	97.49	300,801	1.33
SRR6854684			22,546,871	21,959,502	97.39	300,440	1.33
SRR6854686			25,252,002	24,432,576	96.76	385,458	1.53
SRR6324159		Roots	22,835,231	21,046,995	92.17	479,919	2.10
SRR6324165			21,912,885	20,137,986	91.90	475,748	2.17
SRR6324169			23,642,017	21,363,366	90.36	549,798	2.33
SRR6324170			23,343,403	21,465,924	91.96	577,655	2.47
SRR6324171			23,163,605	21,631,754	93.39	475,735	2.05

## Supplementary Materials

The following are available online at https://www.mdpi.com/2223-7747/9/1/63/s1: Table S1: Full list of DEGs identified between leaves of cultivated and wild cucumber, Table S2: Full list of DEGs identified between roots of cultivated and wild cucumber.
